# Low-profile Visualized Intraluminal Support Junior Device for the Treatment of Intracranial Aneurysms

**DOI:** 10.7759/cureus.1037

**Published:** 2017-02-17

**Authors:** Mihir Gupta, Vincent J Cheung, Peter Abraham, Arvin R Wali, David R Santiago-Dieppa, Brandon C Gabel, Abdulrahman Almansouri, J. Scott Pannell, Alexander A Khalessi

**Affiliations:** 1 Department of Neurosurgery, University of California, San Diego; 2 Department of Neurosurgery, Royal College of Surgeons

**Keywords:** stent-assisted coiling, flow diversion, cerebrovascular disease, lvis jr, endovascular neurosurgery, intracranial aneurysm, fusiform aneurysm, cerebrovascular neurosurgery, angiographic occlusion, endovascular treatment of aneurysm

## Abstract

Objective: Early case series suggest that the recently introduced Low-profile Visualized Intraluminal Support Junior (LVIS Jr.) device (MicroVention-Terumo, Inc., Tustin, CA) may be used to treat wide-necked aneurysms that would otherwise require treatment with intrasaccular devices or open surgery. We report our single-center experience utilizing LVIS Jr. to treat intracranial aneurysms involving 1.8-2.5 mm parent arteries.

Methods: We retrospectively reviewed records of patients treated with the LVIS Jr. device for intracranial aneurysms at a single center. A total of 21 aneurysms were treated in 18 patients. Aneurysms were 2-25 mm in diameter; one was ruptured, while three had recurred after previous rupture and treatment. Lesions were distributed across the anterior (n=12) and posterior (n=9) circulations. Three were fusiform morphology.

Results: Stent deployment was successful in 100% of cases with no immediate complications. Seventeen aneurysms were treated with stent-assisted coil embolization resulting in immediate complete occlusion in 94% of cases. Two fusiform aneurysms arising from the posterior circulation were further treated with elective clip ligation after delayed expansion and recurrence; no lesions required further endovascular treatment. Four aneurysms were treated by flow diversion with stand-alone LVIS Jr. stent, and complete occlusion was achieved in three cases. Small foci of delayed ischemic injury were noted in two patients in the setting of antiplatelet medication noncompliance. No in-stent stenosis, migration, hemorrhage, or permanent deficits were observed. Good functional outcome based on the modified Rankin Scale score (mRS ≤ 2) was achieved in 100% of cases.

Conclusion: Our midterm results suggest that the LVIS Jr. stent may be used for a variety of intracranial aneurysms involving small parent arteries (1.8-2.5 mm) with complete angiographic occlusion, parent vessel preservation, and functional clinical outcomes. This off-label expansion would increase the number of aneurysms amenable to endovascular treatment. Future studies may build upon our experiences with flow diversion and treatment of complex or multiple lesions.

## Introduction

The rapid advancement of endovascular technology has allowed neurointerventionalists to treat complex cerebral aneurysms safely and effectively. Nonetheless, wide-necked and other complex intracranial aneurysms present unique challenges. Specifically, wide-necked aneurysms present a significant risk for prolapse of coils into the parent artery, which can result in devastating thromboembolic or ischemic sequelae [1]. Although sophisticated 3D coil conformations and balloon-assisted techniques allow many wide-necked aneurysms to be effectively treated with stand-alone coil embolization, there remains a large subset of complex, wide-necked aneurysms that can only be satisfactorily treated with the aid of an intracranial stent. Intracranial stents can be further subdivided into devices that are designed to assist coil embolization and those that are designed for stand-alone use as a flow diverter (e.g. Pipeline, Medtronic, Dublin, Ireland).

Since Higashida et al. first reported the placement of an intravascular stent for assistance with coil embolization of a ruptured intracranial aneurysm, stent-coiling techniques have been a cornerstone of the neurointerventionalist's armamentarium [2]. Several successive generations of stents have been developed. One of the most recently released stents is the Low-profile Visible Intraluminal Support device (LVIS and LVIS Jr., MicroVention-Terumo, Inc., Tustin, CA). The LVIS and LVIS Jr. devices are self-expanding, braided, closed-cell nitinol stents with three-pronged, flared ends. The devices include radiopaque proximal and distal markers, as well as three radiopaque helical threads for complete visualization.

At this time, the LVIS and LVIS Jr. stents are approved by the U.S. Food and Drug Administration (FDA) under a Humanitarian Device Exemption (HDE) for unruptured, wide-necked (> 4mm or dome-to-neck ratio < 2), intracranial saccular aneurysms arising from parent vessels 2.5 to 4.5 mm. While the LVIS stent is typically recommended for vessel diameters 3 to 4.5 mm, the LVIS Jr. is recommended for vessel diameters 2.5 to 3 mm [3]. However, the low-profile nature of the LVIS Jr. stent allows for deployment of the device through a 0.017-inch inner diameter microcatheter, which facilitates access of smaller and more tortuous vessels than with earlier stent technologies.

We report our single-institution experience with the use of the LVIS Jr. stent in lesions arising from parent vessels 2.5 mm in diameter or less, both for the purposes of a stent-assisted coil embolization and stand-alone stenting for flow diversion.

## Materials and methods

We retrospectively reviewed records of patients treated with the LVIS Jr. device for intracranial aneurysms involving 1.8 to 2.5 mm parent arteries at a single center. Institutional Review Board approval with waivered patient consent was obtained at the University of California, San Diego. All patient identifying information was removed. This population included 18 consecutive patients with a total of 21 aneurysms over a one-year period between February 2015 and February 2016. The patient population included 13 women (72%) and five men (28%) of mean age 53 years (range 16-80). Median aneurysm dome size was 5.8 mm (range 2 to 25 mm). Only one aneurysm was treated in the setting of an acute rupture. Four aneurysms had undergone previous coiling or clipping treatment; three of these recurrent lesions had a remote history of rupture at the time of LVIS Jr. treatment. Aneurysms were located in both the anterior (n=12) and posterior (n=9) circulations. Three were fusiform in morphology, and one was a dissecting pseudoaneurysm (Table 1).

**Table 1:** Clinical and anatomic characteristics of patients treated using LVIS Jr. for intracranial aneurysms. ACA, anterior cerebral artery; ACOM, anterior communicating artery; ICA, internal carotid artery; MCA, middle cerebral artery; PCA, posterior cerebral artery; PCOM, posterior communicating artery; PICA, posterior inferior cerebellar artery.

Clinical and anatomic characteristics of patients treated using LVIS Jr. for intracranial aneurysms.	
Number of patients	18
Female gender, *n*	13
Mean age ± SD, years	53 ± 18
Number of aneurysms treated	21
Number of stents deployed	20
Aneurysm characteristics, *n*	
Aneurysm status at embolization	
Ruptured at presentation	1
Previously ruptured	3
Recurred after prior coiling	3
Recurred after prior clipping	1
Morphology	
Saccular	17
Fusiform	3
Pseudoaneurysm	1
Location	
ICA	3
ACA	2
MCA	4
ACOM	3
PCOM	2
PCA	1
Basilar tip	4
PICA	2
Aneurysm dome size (mm)	
< 3	3
≥ 3, < 10	16
≥ 10	2
Dome to neck ratio	
≤ 1.5	3
> 1.5, < 2	12
≥ 2	2

Prior to each elective procedure, patients were pretreated with five daily doses of aspirin (325 mg) and clopidogrel (75 mg), including on the day of the procedure. Aspirin and P2Y12 activity levels were checked immediately prior to the procedure to confirm therapeutic platelet inhibition. In the patient who presented with acute aneurysmal subarachnoid hemorrhage, antiplatelet agents were administered post-procedurally. All treatments were performed under general anesthesia with neuromonitoring. After vascular access was obtained, patients were administered a bolus of intravenous heparin (50 units/kg). Guide catheter position was established in either the internal carotid artery or vertebral artery for anterior or posterior circulation aneurysms, respectively.

All aneurysms were treated with a single LVIS Jr. device. Most aneurysms were treated with stent-assisted coiling either by jailing the coiling catheter with the stent using a dual microcatheter technique, or accessing the aneurysm for coiling through the interstices of the stent using a single catheter technique. Coil embolization was completed with bare platinum coils. Multiple types of coils were utilized including: Target 360 coils (Stryker Neurovascular, Fremont, CA); MicroPlex Coil System including HyperSoft, Complex, and Cosmos coils (MicroVention-Terumo, Tustin, CA); CASHMERE coils (Codman Neuro, Raynham, MA); and Axium coils (Medtronic, Dublin, Ireland). In a minority of aneurysms, the size or morphology of the aneurysm was not favorable for coil placement; these were treated with stand-alone stenting for flow diversion. The LVIS Jr. device and coils were delivered through a Headway 17 microcatheter (MicroVention-Terumo, Tustin, CA), Excelsior SL-10 microcatheter (Stryker Neurovascular, Fremont, CA), or Scepter balloon microcatheter (Microvention-Terumo, Tustin, CA).

Following treatment, patients resumed aspirin and clopidogrel on postoperative day one. Elective patients were discharged after overnight observation in the intensive care unit. Further care of patients who presented with aneurysmal rupture was determined by their overall clinical status. Patients were maintained on daily aspirin (325 mg) and clopidogrel (75 mg) for three months, followed by aspirin alone. Postoperative angiograms were typically performed three to six months after intervention to assess for any aneurysm residual or recurrence and to determine the quality of the treatment. All angiograms were reviewed and scored by a board-certified neuroradiologist blinded to the type of intervention. Angiographic occlusion was graded by consensus scale for stent-assisted coiling or modified Raymond-Roy score for flow diversion [4-5]. Good functional outcome was defined as mRS ≤ 2.

## Results

**Figure 1:**
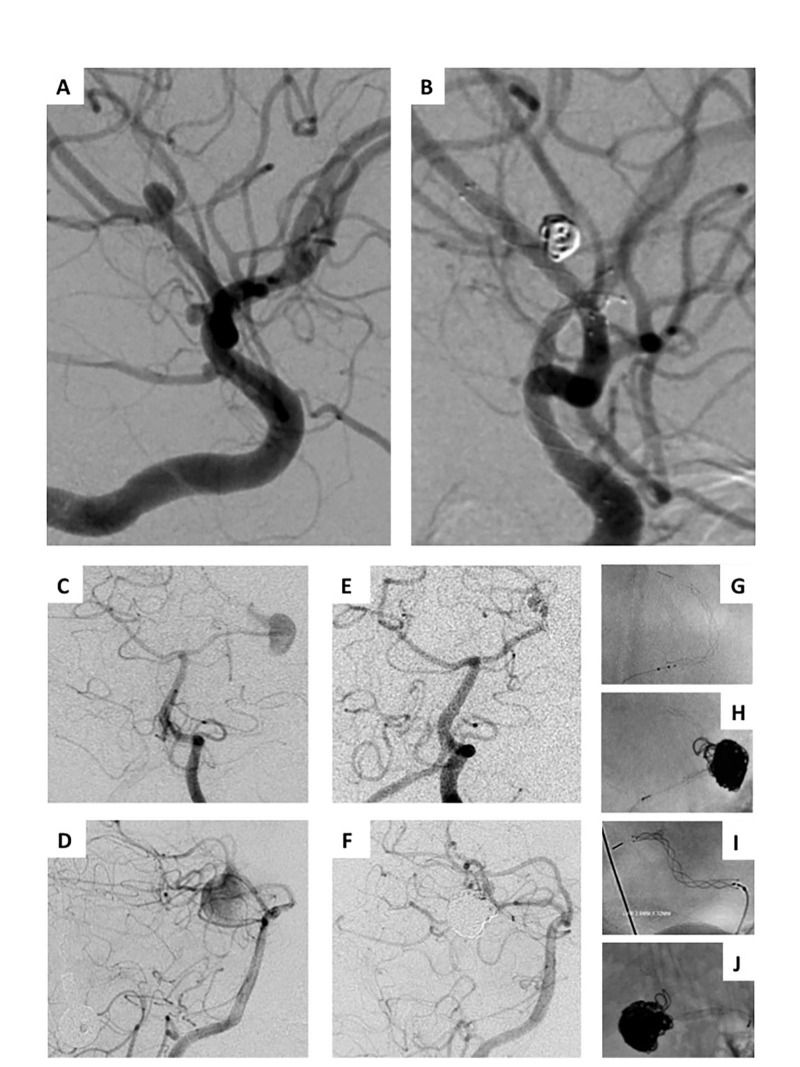
Stent-assisted coiling and flow diversion with LVIS Jr. (A) Oblique lateral right anterior circulation angiogram of a patient with right MCA M2 bifurcation aneurysm, right ICA terminus, and right PCOM blister aneurysms. (B) Complete occlusion of the MCA bifurcation aneurysm after stent-assisted coiling, and complete occlusion of the PCOM and ICA terminus aneurysms after flow diversion. (C, D) Posterior-anterior and lateral posterior circulation angiograms of a patient with a giant fusiform left PICA aneurysm. (E, F) Complete obliteration of the aneurysm after stent-assisted coiling. (G-J) Unsubtracted images showing stent and coil reconstruction of parent vessel. MCA, middle cerebral artery; ICA, internal carotid artery; PCOM, posterior communicating artery; PICA, posterior inferior cerebellar artery.

Stent deployment was successful in 100% of cases (20/20) with no immediate complications. Seventeen aneurysms were treated with stent-assisted coil embolization with immediate complete occlusion in 94% (16/17) and consensus grade 1 (90% or greater occlusion) in one patient (Table 2). Four aneurysms were treated by flow diversion with stand-alone stenting, and complete occlusion at last follow-up was achieved in three of these cases (Figure 1).

**Table 2:** Stent implantation procedure characteristics and outcomes. †Defined as modified Rankin Scale score ≤ 2 at last follow-up.

Stent implantation procedure characteristics and outcomes.		
Stent size (mm)		
2.5 x 13		2
2.5 x 17		3
2.5 x 23		3
2.5 x 34		1
3.5 x 18		7
3.5 x 23		4
Successful deployment		20 (100%)
Treatment modality		
Stent-assisted coiling		17
Stand-alone stenting		4
Good functional outcome^†^		18 (100%)
Death or major adverse event		0 (0%)
Minor adverse event		2 (11%)
Further treatment required		2 (11%)

Over a median of eight months of follow-up, good functional outcome was achieved in 100% of cases (20/20). Small foci areas of diffusion-weighted imaging positivity consistent with embolic ischemic events were noted in two patients on delayed follow-up magnetic resonance imaging (MRI): one was asymptomatic, and another experienced transient neurologic deficit in the setting of medication noncompliance. No cases of in-stent stenosis, stent migration, hemorrhage, or permanent neurologic deficits were observed. No patients received additional endovascular treatment. No new neurologic deficit was noted on follow-up.

**Figure 2:**
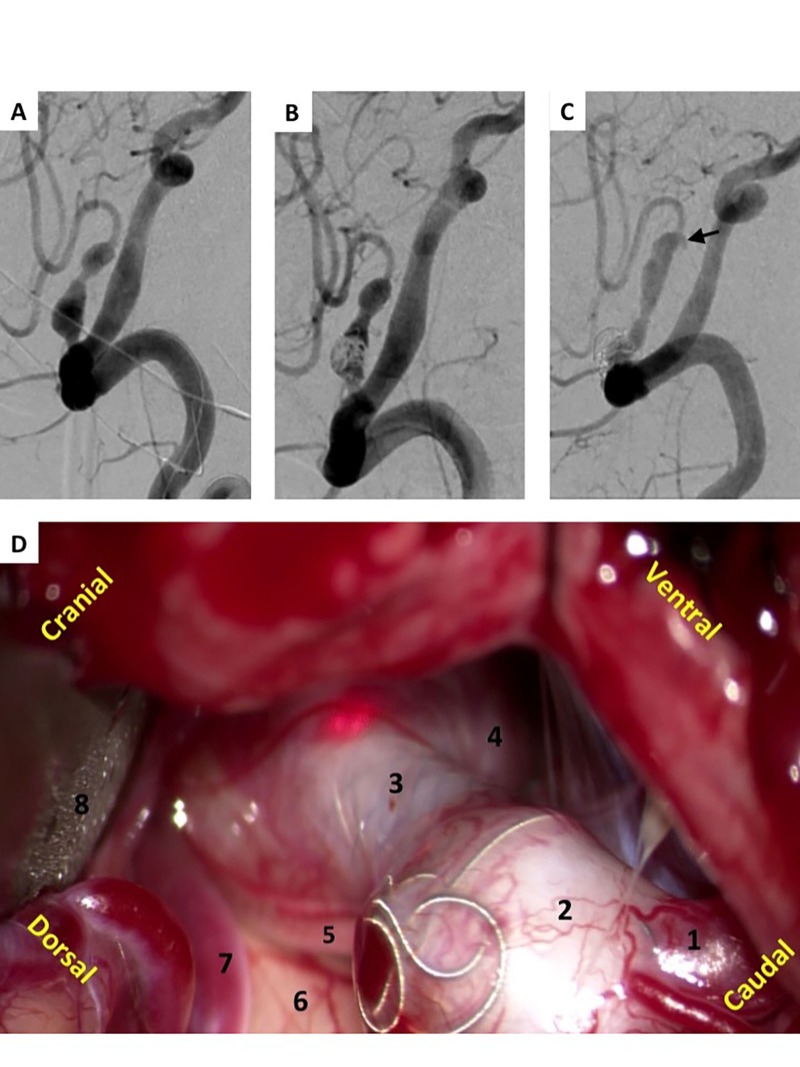
Stent-assisted coiling and subsequent clip ligation of PICA aneurysm. Lateral posterior circulation angiogram demonstrating ruptured right PICA aneurysm with fusiform dissection, (A) before and (B) after stent-assisted coiling with LVIS Jr. (C) Six-month lateral posterior circulation angiogram revealed delayed type 1b endoleak with dissection extending beyond stent (arrow). (D) Intraoperative photograph of far lateral exposure of delayed recurrent lesion demonstrating (1) proximal PICA, (2) previously coiled aneurysm, (3) stent, (4) recurrent aneurysm, (5) glossopharyngeal nerve, (6) posterior lateral surface of medulla, (7) distal PICA, and (8) retracted cerebellum. PICA, posterior inferior cerebellar artery.

Two fusiform aneurysms arising from the posterior circulation required additional elective open surgical reconstruction after asymptomatic recurrence was noted on follow-up angiography. One of the fusiform aneurysms was a ruptured, dissecting, posterior inferior cerebellar artery (PICA) aneurysm, which extended beyond the distal stent construct into the PICA (Figure 2). The other fusiform lesion was an unruptured posterior cerebral artery (PCA) aneurysm in which fusiform recurrence developed. Following open surgical reconstruction, both patients recovered without permanent neurologic deficits.

## Discussion

Our initial experience with the LVIS Jr. stent demonstrated that the technology can be safely used in the treatment of intracranial aneurysms. Notably, LVIS Jr. can be safely deployed in parent vessels under 2 mm providing an endovascular treatment alternative for wide-necked aneurysms in distal vascular distributions that would previously be reserved for microsurgical clipping, including M2 middle cerebral artery (MCA), PICA, and pericallosal anterior cerebral artery (ACA) aneurysms. We observed no stent-related complications in patients who were compliant with dual antiplatelet therapy.

The LVIS Jr. stent possesses multiple technical advantages over existing intracranial stents. First, the 0.017-inch inner diameter of the stent deployment microcatheter is the smallest available stent delivery system, which is also the most commonly used catheter for coil embolization. We employed the widest variety of coils from any published series to date and observed no incompatibility. Furthermore, we found that the LVIS Jr. can be deployed through a conventional 0.0165-inch Stryker SL-10 microcatheter on an off-label basis. Second, the LVIS Jr. has three radiopaque strands along its entire length as well as three proximal and distal radiopaque markers, allowing the operator to visualize its expansion during deployment. Other intracranial stents only have markers at the proximal and distal tines.

Limitations of this study include its retrospective nature and the relatively small number of patients treated. However, our clinical results compare favorably to those observed in recent published series of LVIS Jr. [3,6-16]. The majority of published series include only short-term follow-up. Only two prior studies demonstrate midterm follow-up results over a duration similar to ours. Shankar et al. describe 100 patients with saccular aneurysms with a median of 12 months of follow-up, but had higher angiographic and clinical complications than observed in other series. Complications were not significantly associated with any identifiable factors in multivariate analysis, though it is possible that the less aggressive anticoagulation regimen of three months of aspirin (81 mg) and clopidogrel (75 mg), followed by only one medication, contributed to the high rates of thrombus formation, thromboembolic phenomena, and neurologic deficit [16]. Alghamdi et al. describe 40 patients followed for a median of 12 months with more favorable outcomes, but lesions in this series were only 2 to 13 mm in size [6].

Notably, we describe the first cases to use LVIS Jr. for flow diversion, as well as the use of a single stent to treat multiple aneurysms arising from the same vessel. Due to its braided design, small interstices (cell size ~ 1.5 mm), and neck coverage surface area of approximately 18%, LVIS Jr. has flow-diversion properties that enable stand-alone treatment for aneurysms that are too small to coil or that arise from vessels that are too small to accommodate a traditional flow diverter. Although the higher area of metal coverage raises the possibility of in-stent stenosis due to neointimalization, this has only been observed in a minority of published series [3,6].

## Conclusions

Our initial, single-center experience with the LVIS Jr. stent demonstrates this treatment modality is safe for use in parent vessel sizes below the suggested diameter with midterm clinical results suggesting durable aneurysm occlusion and absence of parent vessel compromise. Safe deployment in vessels under 2 mm expands the range of aneurysms treatable via an endovascular approach. LVIS Jr. radiopacity was sufficient to allow precise placement in fusiform cases, and surface coverage was sufficient as a stand-alone flow-diversion treatment for uncoilable blister aneurysms arising from small parent vessels.

## References

[1] Lee YJ, Kim DJ, Suh SH (2005). Stent-assisted coil embolization of intracranial wide-necked aneurysms. Neuroradiology.

[2] Higashida RT, Smith W, Gress D (1997). Intravascular stent and endovascular coil placement for a ruptured fusiform aneurysm of the basilar artery. Case report and review of the literature. J Neurosurg.

[3] Cho YD, Sohn CH, Kang HS (2014). Coil embolization of intracranial saccular aneurysms using the Low-profile Visualized Intraluminal Support (LVIS™) device. Neuroradiology.

[4] Meyers PM, Schumacher HC, Higashida RT (2010). Reporting standards for endovascular repair of saccular intracranial cerebral aneurysms. J Neurointerv Surg.

[5] Cekirge HS, Saatci I (2016). A new aneurysm occlusion classification after the impact of flow modification. AJNR Am J Neuroradiol.

[6] Alghamdi F, Mine B, Morais R (2016). Stent-assisted coiling of intracranial aneurysms located on small vessels: midterm results with the LVIS Junior stent in 40 patients with 43 aneurysms. Neuroradiology.

[7] Behme D, Weber A, Kowoll A (2015). Low-profile Visualized Intraluminal Support device (LVIS Jr) as a novel tool in the treatment of wide-necked intracranial aneurysms: initial experience in 32 cases. J Neurointerv Surg.

[8] Feng Z, Fang Y, Xu Y (2016). The safety and efficacy of low profile visualized intraluminal support (LVIS) stents in assisting coil embolization of intracranial saccular aneurysms: a single center experience. J Neurointerv Surg.

[9] Feng Z, Li Q, Zhao R (2015). Endovascular treatment of middle cerebral artery aneurysm with the LVIS Junior stent. J Stroke Cerebrovasc Dis.

[10] Feng Z, Zhang L, Li Q (2015). Endovascular treatment of wide-neck anterior communicating artery aneurysms using the LVIS Junior stent. J Clin Neurosci.

[11] Fiorella D, Arthur A, Boulos A (2016). Final results of the US humanitarian device exemption study of the low-profile visualized intraluminal support (LVIS) device. J Neurointerv Surg.

[12] Grossberg JA, Hanel RA, Dabus G (2016). Treatment of wide-necked aneurysms with the Low-profile Visualized Intraluminal Support (LVIS Jr) device: a multicenter experience. J Neurointerv Surg.

[13] Mohlenbruch M, Herweh C, Behrens L (2014). The LVIS Jr. microstent to assist coil embolization of wide-neck intracranial aneurysms: clinical study to assess safety and efficacy. Neuroradiology.

[14] Poncyljusz W, Bilinski P, Safranow K (2015). The LVIS/LVIS Jr. stents in the treatment of wide-neck intracranial aneurysms: multicentre registry. J Neurointerv Surg.

[15] Samaniego EA, Abdo G, Hanel RA (2016). Endovascular treatment of PICA aneurysms with a Low-profile Visualized Intraluminal Support (LVIS Jr) device. J Neurointerv Surg.

[16] Shankar JJ, Quateen A, Weill A (2016). Canadian registry of LVIS Jr for treatment of intracranial aneurysms (CaRLA). J Neurointerv Surg.

